# Cross-talk between immunity and behavior: insights from entomopathogenic fungi and their insect hosts

**DOI:** 10.1093/femsre/fuae003

**Published:** 2024-02-10

**Authors:** Wei Zhang, Xuanyu Chen, Ioannis Eleftherianos, Amr Mohamed, Ashley Bastin, Nemat O Keyhani

**Affiliations:** National Key Laboratory of Green Pesticide, Key Laboratory of Green Pesticide and Agricultural Bioengineering (Ministry of Education), Guizhou University, Guiyang, Huaxi District 550025, China; National Key Laboratory of Green Pesticide, Key Laboratory of Green Pesticide and Agricultural Bioengineering (Ministry of Education), Guizhou University, Guiyang, Huaxi District 550025, China; Department of Biological Sciences, The George Washington University, Washington, DC 20052, United States; Department of Entomology, Faculty of Science, Cairo University, Giza 12613, Egypt; Research fellow, King Saud University Museum of Arthropods, Plant Protection Department, College of Food and Agricultural Sciences, King Saud University, Saudi Arabia; Department of Biological Sciences, The George Washington University, Washington, DC 20052, United States; Department of Biological Sciences, University of Illinois, Chicago, IL 60607, United States

**Keywords:** innate immunity, behavior, chemicals, ligand receptors, cross-talk, entomopathogenic fungi

## Abstract

Insects are one of the most successful animals in nature, and entomopathogenic fungi play a significant role in the natural epizootic control of insect populations in many ecosystems. The interaction between insects and entomopathogenic fungi has continuously coevolved over hundreds of millions of years. Many components of the insect innate immune responses against fungal infection are conserved across phyla. Additionally, behavioral responses, which include avoidance, grooming, and/or modulation of body temperature, have been recognized as important mechanisms for opposing fungal pathogens. In an effort to investigate possible cross-talk and mediating mechanisms between these fundamental biological processes, recent studies have integrated and/or explored immune and behavioral responses. Current information indicates that during discrete stages of fungal infection, several insect behavioral and immune responses are altered simultaneously, suggesting important connections between the two systems. This review synthesizes recent advances in our understanding of the physiological and molecular aspects influencing cross-talk between behavioral and innate immune antifungal reactions, including chemical perception and olfactory pathways.

## Teaser

This study reviews recent articles suggesting that insect behavioral and innate immune responses are simultaneously regulated during discrete stages of fungal infection and highlights the biological significance of this cross-talk.

## Introduction

Insects comprise ~67% of the known global fauna, with the Insecta including at least one million species (Stork 2018). Insects are integral to the functioning of almost all ecosystems (Crespo-Pérez et al. 2020). Although most insects obtain nutrients from plant leaves, stems, roots, and/or fruits, numerous carnivorous, omnivorous, and/or mycophagous insects exist in high numbers in various environments, particularly social insects. Despite being a major source of food and hence preyed upon by different animals, including amphibians, reptiles, birds, and mammals, over 60% of insect fatalities in nature are attributed to fungal mycoses (Cole 2012).

Entomopathogenic fungi represent a highly diverse group with ~1600 known species in 90 genera, whose ability to infect insects has evolved multiple times in different lineages. Genera and species of entomopathogenic fungi can be found distributed within the Ascomycota, Basidiomycota, Blastocladiomycota, Chytridiomycota, Entomophthoromycotina (Zoopagomycota), Microsporidia, and Mucoromycota, as well as within the fungus-like Oomycota (Hajek and St. Leger 1994, Boomsma et al. 2014). Entomopathogenic fungi infect over 18 orders of insects at all developmental stages, from eggs to adults (Araújo and Hughes 2016). Most Ascomycete entomopathogenic fungi, including *Metarhizium* and *Beauveria* species, infect insects via attachment and direct hyphal penetration of the chitinous exoskeleton. Depending upon the species, this process may involve the production of cuticle-penetrating appressoria, akin to those found on certain plant pathogenic fungi, but hyphal penetration without clearly defined appressoria also occurs (Ortiz-Urquiza and Keyhani 2013, Chethana et al. 2021). Subsequent to penetration, growth in the hemocoel follows, accompanied by the production of immune-evading free-floating yeast-like cells, termed hyphal bodies or *in vivo* blastospores (Wang et al. 2023). Several other related entomopathogens (outside the Hypocreales) use a broadly similar strategy, i.e. produce *in vivo* protoplasts (cells without cell walls, Oomycetes) or hyphae (Chytridiomycetes and Entomophthoromycetes) during insect host colonization (Elya and De Fine Licht 2021, Sacco and Hajek 2023). Postcolonization, some entomopathogenic fungal species are biotrophic or even obligate, producing spores from living or dying hosts that subsequently infect other hosts; however, most are hemibiotrophic, sporulating on dead hosts (Castrillo et al. 2005). Apart from transmission to other host insects directly, the spores of hemibiotrophic species can lie dormant on leaves or in the soil, grow saprophytically in the presence of suitable nutrients, or form mutualistic associations with plants, either in the rhizosphere as epiphytes or as endophytes of certain plant hosts (Araújo and Hughes 2016, Dara 2019). The diversity of species, broad distribution, and specialized infection routes have given rise to a range of prolonged, complex, varied, and intriguing molecular, physiological, and ecological interactions between entomopathogenic fungi and insects.

**Figure 1.**
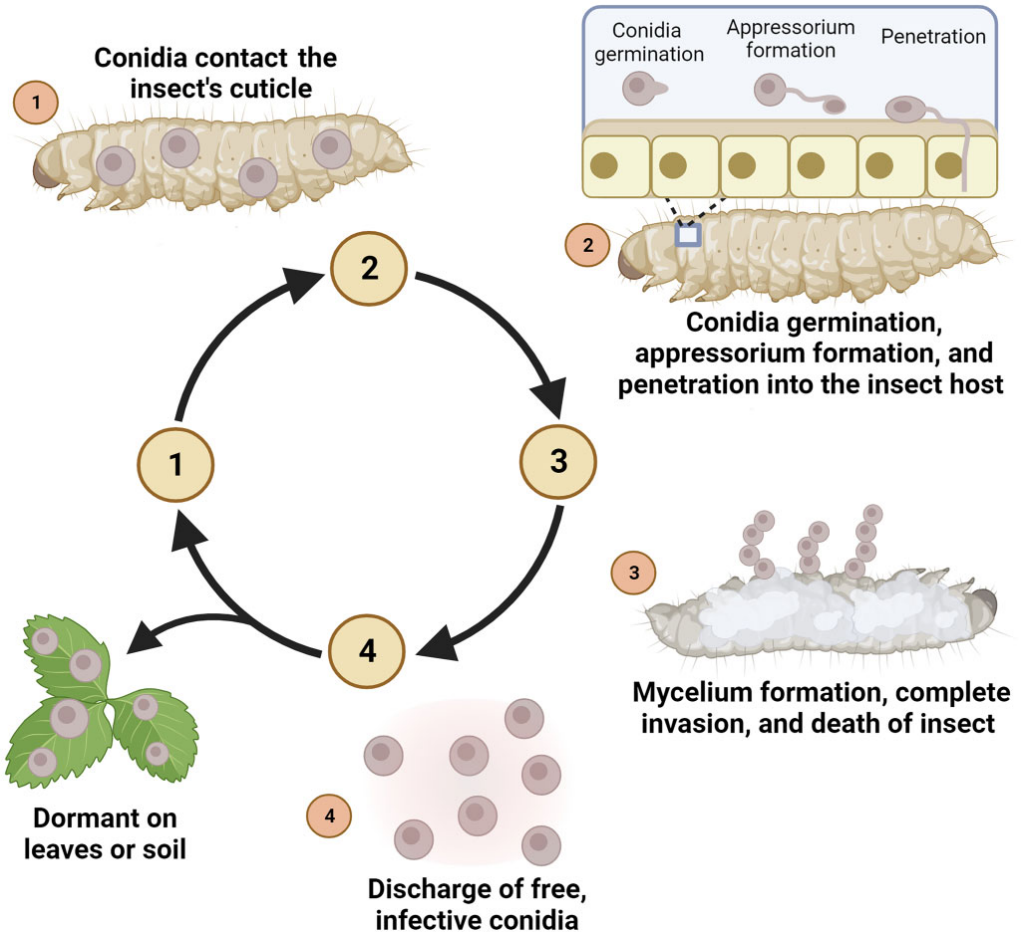
Simplified infection processes of entomopathogenic fungi to insect hosts. The processes of fungal infection of insect hosts can be divided into several stages: (1) conidial contact and subsequent adhesion to the host cuticle, (2) conidial germination, germ tube/appressorium formation, and penetration into the insect host, (3) hyphal body formation, proliferation in within the insect hemocoel and surrounding tissues, hyphal extension outwards, and death of the insect, and (4) fungal sporulation and shedding from the cadaver.

Recent advances in the development of tools for genetic, genomic, molecular, bioinformatic, biochemical, and ecological analyses have significantly augmented our understanding of the mechanisms governing interactions between insects and pathogenic fungi (Hajek and St. Leger 1994, Roy et al. 2006, Ortiz-Urquiza and Keyhani 2013, Butt et al. 2016, Wang and Wang 2017, Wang et al. 2023, Hong et al. 2023a, Liu et al. 2023a). Most research has predominantly concentrated on several genera of Ascomycota, e.g. *Beauveria, Metarhizium*, and *Cordyceps*, as well as members of the Entomophthorales, several of which have been globally commercialized for pest control (de Faria and Wraight 2007, Wang and Feng 2014, Bamisile et al. 2021). The fungal–insect physiological and ecological interaction unfolds across several broad stages during the fungal infection of susceptible hosts (Fig. 1). These stages include: (1) precontact of the fungal spore (or mycelium) prior to attachment to the host exoskeleton. At this stage, entomopathogenic fungi produce volatile organic compounds (VOCs) that can induce behavioral or physiological responses, influencing the infection process (Baverstock et al. 2009). (2) Cuticle contact: entomopathogenic fungi attach, germinate, and subsequently penetrate the host exoskeleton through the production of infection-related substances. Some fungal components can induce the production of antimicrobial substances in the cuticle or prompt grooming, thermoregulation, or heat-seeking behavior to reduce spore infection on the surface (Roy et al. 2006). (3) Internal growth within the host hemocoel and other tissues; host cellular and humoral immune responses are activated, and/or behavioral responses may change. Entomopathogenic fungi adapt to host antimicrobial responses by altering their cell wall components, producing toxins and metabolites to evade immune responses, and/or manipulating host behavior (Qu and Wang 2018). Infection of host tissues (beyond the hemocoel) ultimately leads to host death. (4) Penetration (outwards) from inside the host, followed by growth and sporulation of the fungus on the surface of the insect cadaver. Within the context of the latter, some social insects exhibit sanitation behaviors, actively removing or treating the corpses of individuals that died from fungal infection to minimize the spread of infection (Fan et al. 2012, Sun and Zhou 2013).

**Figure 2.**
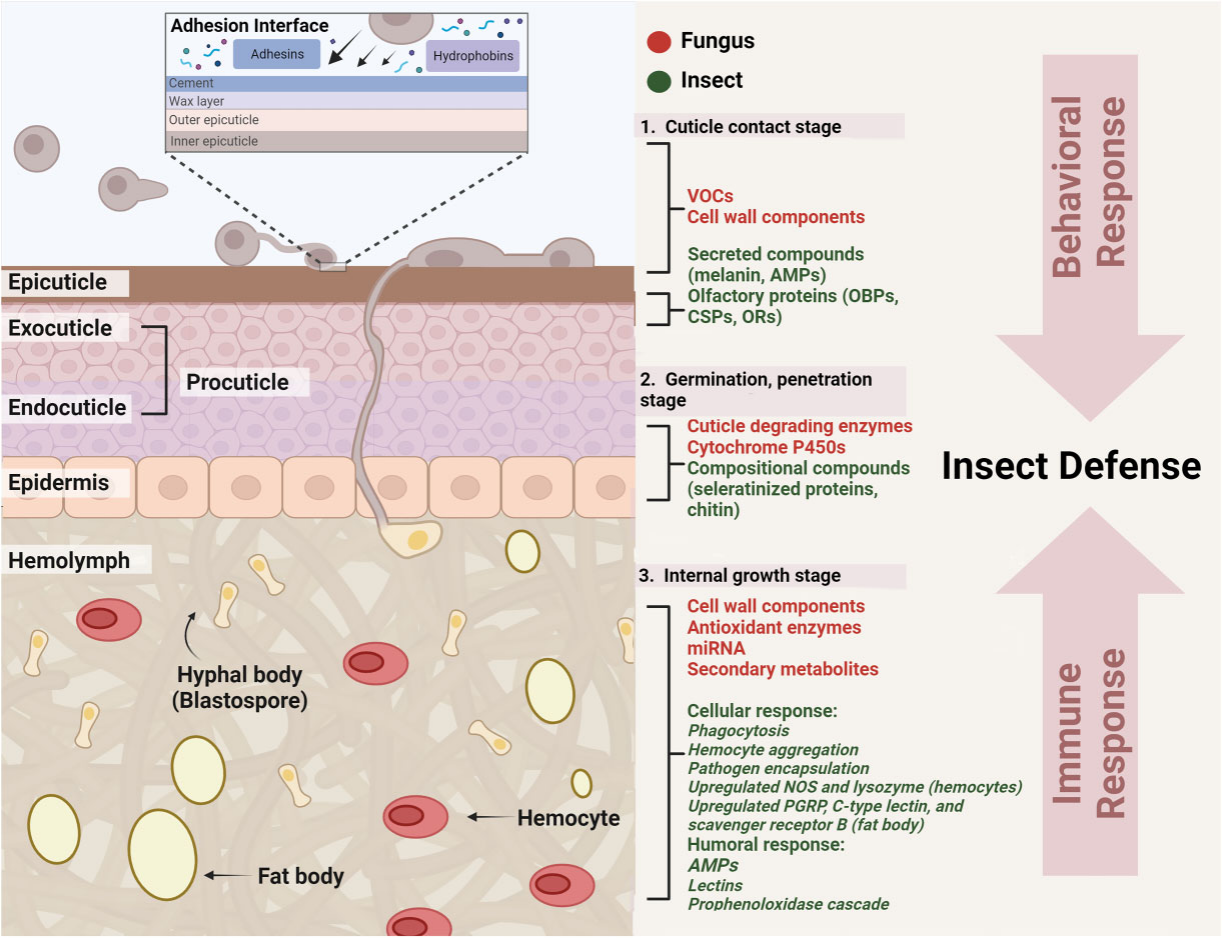
Fungal and host factors that mediate insect behavioral and immune responses. Before and during the cuticle contact/adhesion stage, fungal released VOCs act as immune/behavioral elicitors on host olfactory proteins; fungal hydrophobins, adhesins, and cell wall components mediate attachment, and the insect cuticle acts as the initial antimicrobial barrier through the production of hydrocarbons, melaninization, and/or antimicrobial peptides (AMPs). As spores germinate and penetrate the host cuticle, fungal-produced cuticle-degrading enzymes hydrolyze host chitin and sclerotized protein cuticle. Within the hemocoel, fungal secondary metabolites, miRNAs, effectors, enzymes, and cell wall components are released that may be recognized and affect immune/behavioral responses through action on host (PRPs), Toll/Imd other immune pathways, and/or ligand binding proteins (chemosensory and odorant binding proteins/receptors) to modulate insect behavior or immunity.

Several studies have shown that innate immunity and behavioral responses can occur simultaneously at various stages of infection and that these responses can influence each other. The central nervous system (CNS) is likely to be involved in immunity (Lampron et al. 2013), and conversely, key immune cells and tissues, such as hemocytes and the fat body, may also contribute to the behavioral responses of insects (Kamimura et al. 2020). Recent evidence suggests that the insect cuticle may function as a platform that potentially directs both behavioral and immune responses (Ortiz-Urquiza and Keyhani 2013). Generally, both behavioral immune and innate immune responses necessitate the recognition of pathogen-derived signals, presumably via specific soluble and/or membrane-bound ligand–receptor proteins. This recognition leads to signal transduction, output, and ultimately the induction of immune and behavioral responses (Fig. 2). In typical innate immunity pathways, insects launch cellular and humoral immune functions via recognition of pathogen cues that act to initiate distinct signal transduction cascades that subsequently activate physiological responses. Insect immune reactions can include the production of antimicrobial enzymes and peptides, phagocytosis by immune cells, melanization, and nodule formation (Zhang et al. 2021a). Significant progress has been made with respect to the recognition of pathogen markers, e.g. cell wall glucan, effectors, and other pathogen-associated molecular patterns (PAMPs), via host signal transduction pathways such as the Toll, IMD (immune deficiency), and JAK/STAT (Janus kinase/signal transducers and activators of transcription), that cooperate to initiate an immune response (Qu and Wang 2018, Zhang et al. 2021b). Although far less understood, several signals and proteins related to behavioral immunity have also been identified (Roy et al. 2006). In addition, several compounds and proteins that function in behavior have been described to function in immunity, and here, we review the current reports examining the cross-talk between behavioral (including social immunity) and innate/humoral immune responses during fungal infection.

Our limited understanding of host defense systems, particularly in terms of their origin and evolution with respect to fungal pathogens, has significantly impeded our ability to fully understand the complex interactions between insects and pathogenic fungi. Unraveling the mechanisms behind the cross-talk between behavior and immunity is crucial, as it holds significant implications in immunology, behavior, ecology, and evolution and can contribute new insights to the development of the broad field of host–pathogen interactions. This area of study likely represents an emerging field that reflects the evolutionary selection pressures resulting from the ongoing “arms race” between insects and fungal pathogens. Additionally, it is widely acknowledged in terms of practical applications that fungal-based “bio-pesticides” often exhibit slow mortality rates and inconsistent efficacy in field application for control of agricultural, invasive, human health-relevant, and other insect pests, potentially due to the insect behavioral immune responses, innate immune responses, or cross-talk between these defenses. The development and use of more potent fungal biological control agents for pest management, in particular, may greatly benefit from an understanding of the interactions between immune and behavioral responses.

## Cross-talk between olfaction, behavior, and immunity: before contact of host cuticle with entomopathogenic fungi

Before entomopathogenic fungi engage, i.e. attach to the host cuticle, insects may engage in avoidance behaviors to minimize contact (and hence infection). For example, the seven-spotted ladybird, *Coccinella septempunctata*, can detect and avoid leaves and soil containing *Beauveria bassiana* (Ormond et al. 2011). Mole crickets also avoid contact with soil harboring *B. bassiana* (Thompson and Brandenburg 2013). Such environmental avoidance behaviors can impact fungal pesticide efficacy. The Japanese beetle, *Popillia japonica* (Villani et al. 1994), and termites (Staples and Milner 2000) exhibit avoidance behaviors toward soil containing entomopathogenic fungi, likely providing one mechanism that can diminish the effectiveness of the application of fungal pesticides toward these insects. Beyond triggering behavioral effects, several VOCs emitted by fungi can induce physiological effects on insects without direct contact. The termite *Macrotermes michaelseni* can discriminate between virulent and avirulent strains of *Metarhizium anisopliae* and *B. bassiana* based on emitted VOC profiles (Mburu et al. 2011). However, arthropods vary in their ability to detect and avoid entomopathogenic fungi based on species, developmental stage, sex, and ecological location (e.g. soil, rhizosphere, and plant phylloplane) of the fungus (Baverstock et al. 2009). For instance, *Agriotes obscurus* larvae, but not male adults, avoid *M. anisopliae* (Janmaat et al. 2022). Flower beetles avoid *B. bassiana* when present on leaves but not in soil (Meyling and Pell 2006), whereas Japanese beetle larvae avoid soil-borne *M. anisopliae* (Villani et al. 1994). Some social insect species, such as the termites *Reticulotermes flavipes, M. michaelseni, Zootermopsis augusticollis*, and the ants *Acromyrmex striatus*, and *Formica rufa*, avoid fungal-infected areas or nestmates (Myles 2002, Mburu et al. 2009, Liu et al. 2019a). However, other species, such as *Myrmica ruba*, do not avoid fungal contamination, including *M. brunneum* found in surrounding areas (Pereira et al. 2021). In some instances, social insects can detect but react differentially to diseased workers outside versus inside the nest, with health-detectable cues tunable within the social context (e.g. *M. ruba* infected by *M. anisopliae*) (Leclerc and Detrain 2016). *Reticulitermes flavipes* termite workers can even be reintegrated into a colony after infection with *Metarhizium* fungi (Moran et al. 2022). Founding queens of the ant *Formica selysi* appear attracted to soil “contaminated” with *Beauveria* or *Metarhizium* (Bruetsch et al. 2014). Whether this represents parasite manipulation or benefits the ant via the selection of suitable nesting sites remains unclear. Nematophagous fungi, such as *Pochonia chlamydosporia* (intriguingly with a similar genome composition as *Metarhizium*), produce VOCs that are active against both nematodes and insects, indicating potential coevolutionary resemblances toward different hosts by fungal pathogens (Lozano-Soria et al. 2020).

Various VOCs have been identified from entomopathogenic fungi, including fatty and other acids, esters, terpenes, aldehydes, ketones, alcohols, and other organic compounds (Bojke et al. 2018). These compounds represent both primary and secondary metabolites and are synthesized through diverse biochemical pathways, with esters, acids, and terpenoids being the most predominant (Jeleń and Wasowicz 1998). Some VOCs have been shown to affect insect behavior. For example, the terpenoid geosmin, prevalent in many fungi, along with benzaldehyde, induces avoidance behavior in the fruit fly *Drosophila melanogaster* (Störtkuhl et al 2005). Fruit flies can detect dimethyl trisulfide and 2-phenylethanol, avoiding ovipositing in contaminated areas (Holighaus and Rohlfs 2016). Octanol and hexanol in *M. anisopliae* and other fungi attract sand fly females (Machado et al. 2015). Some mushrooms have been reported to repel fungivorous insects by producing 1-octen-3-ol (Holighaus et al. 2014). 1-Octen-3-ol can also modulate insect behavior by acting as a repellent or an attractant to different insects (Fäldt et al. 1999, El Jaddaoui et al. 2023). Although 1-octen-3-ol is also produced by entomopathogenic fungi, its potential role in mediating insect behavior and immunity remains unknown. Intriguingly, 1-octen-3-ol can serve as an inhibitor of fungal spore germination, indicating its potential role in microbial ecology through the reduction of intraspecific or interspecific competition when spore concentrations are high (Chitarra et al. 2004, Hummadi et al. 2022).

As opposed to repelling, certain entomopathogenic fungi are able to attract insect hosts, indicating a distinctly different evolutionary pathway. The green peach aphid, *Myzus persicae*, is attracted by *B. bassiana* conidia (Geedi et al. 2023), and *M. brunneum* rhizosphere interactions with cabbage (*Brassica oleracea*) appear to manipulate plant VOC production to attract herbivorous insects (Cotes et al. 2020). In addition to short- and medium-chain volatiles, fungi produce a diverse mix of terpenes, such as γ-gurjunen by *B. bassiana*, β-elemene by *Isaria fumosorosea*, and α-farnesene by *Hirsutella danubiensis* (Bojke et al. 2018). Essential oils containing terpenes exhibit antimicrobial and antioxidant properties (Bozin et al. 2007, Diao et al. 2013). The dominant fatty acid, palmitic acid (C16:0), in *B. bassiana, Batkoa* spp., *I. fumosorosea*, and *Metarhizium flavoviride* plays crucial roles in intermediary metabolism (Bojke et al. 2018) and affects the growth of the cotton bollworm, *Helicoverpa armigera* (Satyan et al. 2009). Additionally, certain VOCs, such as 3-methyl-1-butanol, and compounds with limited volatility, including ochratoxin, aflatoxins, penicillic acid, and various *Fusarium* toxins and derivatives, exhibit cytotoxic properties toward insect cells (Holighaus and Rohlfs 2016). These findings suggest that fungal VOCs or nonvolatiles likely participate in behavioral, toxicity, and immune responses (Fig. 2). However, the underlying mechanisms driving these actions are poorly characterized. Biotic and abiotic environmental factors are known to trigger the differential production of metabolites, which likely results in altered VOC profiles. These altered profiles could induce varying behavioral responses in different target insects (Bennett et al. 2012). Despite the potential significance of these interactions, they remain relatively underexplored in terms of underlying genetic and biochemical mechanisms, not only due to their often being overlooked but potentially to difficulties in experimental models and approaches to examine these interactions.

However, several important insights have been gained. Gas phase 1-octen-3-ol acts on *D. melanogaster* dopaminergic neurons in laboratory tests, even at low concentrations, causing neurotoxic and cytotoxic effects (Morath et al. 2012), indicating that dopamine and dopamine receptors may play a role in mediating immunity and behavioral responses. Phenethyl ethanol, produced by *M. anisopliae*, has been shown to affect both avoidance behavior and immune inhibition in locusts. Moreover, a specific locust odorant binding protein (OBP; LmigOBP11) has been identified as a mediator of 2-phenylethanol detection, influencing insect innate immune responses in the hemolymph (Zhang et al. 2023a). Intriguingly, LmigOBP11 inhibits insect immune responses and is upregulated during *M. anisopliae* infection, suggesting that the fungus manipulates OBP expression to enhance successful mycosis. These findings support the emergence of a field exploring molecular cross-talk between insect detection of fungal VOCs via odorant/ligand binding pathways, subsequent avoidance behaviors, and immune activation. Identifying additional insect and fungal molecular determinants involved in VOC production (fungal) and detection (insect) associated with specific behaviors will likely reveal crucial layers to these interactions. The notion that the fungal pathogen exploits host detection to suppress immunity adds a novel dimension to the coevolutionary arms race between these organisms.

## Cross-talk: during fungal cuticle contact and penetration

Insects possess mechanisms for detecting and initiating hygienic behaviors when entomopathogenic fungal spores attach to their cuticle. Self-grooming increases in *F. selysi* ant workers exposed to *M. brunneum* (Tragust et al. 2013). Other ant species, such as the red imported fire ant, *Solenopsis invicta* (Qiu et al. 2014), the formic acid spraying Asian ant, *Lasius japonicus* (Okuno et al. 2012), and the carnivorous ant, *Platythyrea punctata* (Westhus et al. 2014), also display nestmate grooming behaviors in response to detection of fungal pathogens. In termites, alarm pheromone can act as a trigger for hygienic grooming, e.g. in *R. flavipes* infected with *Metarhizium* (Bulmer et al. 2019). This behavior involves not only physically removing spores but also producing antimicrobial proteins, lipids, and metabolites in the epicuticle. In the cotton aphid (Kim and Roberts 2012) and diamondback moth (Vandenberg et al. 1998), infection by *B. bassiana* and/or *Lecanicillium attenuatum* speeds up instar development, potentially as a way to elude infection into the next life stage by shedding infected structures. Some insects, including grasshoppers (*Schistocerca gregaria*), exhibit “behavioral fever” by raising their body temperature through sun basking to combat *Metarhizium* infection (Ouedraogo et al. 2004). These behaviors are critical for some insects, resulting in significant reductions in infecting conidial numbers and thus improving infected insect survival rates. Physiological immune responses are also initiated at this stage, typically involving (i) hemocytes and fat bodies migrating to the wound site to promote repair and prevent/reduce infection; (ii) melanization to sequester and kill the invading microbe; (iii) induction of prophenoloxidase and other immune related enzymes; and/or (iv) antimicrobial peptide (AMP) production within the hemocoel and potentially on the cuticle (Qu and Wang 2018). Although only limited information is available, behavioral and immunological responses may compete with each other. For example, in termites, intragrooming behaviors in response to *Metarhizium* exposure lead to reduced AMP production, indicating optimization or trade-off effects between behavioral and immune responses (Liu et al. 2019b).

Depending on the insect species, the cuticle is considered to contain an outermost “waxy layer” with various long-to-mid-chain hydrocarbons, including alkanes, alkenes, and their methyl-branched derivatives, fatty acids and esters, alcohols, ketones, and aldehydes, as well as minor components, including triacylglycerols, epoxides, and ethers (Chung and Carroll 2015, Golian et al. 2022) (Fig. 2). Some insect epicuticular lipids are useful substrates for entomopathogenic fungi and contribute to crucial prepenetration events during infection (Jarrold et al. 2007, Zhang et al. 2012, Pedrini et al. 2013). Under the waxy layer, the highly sclerotized portion of the cuticle consists of chitin-cross-linked tanned proteins, with fungal chitinases long considered important virulence factors (Charnley 2003). Insect cuticular constituents play crucial roles in environmental adaptation and act as information and communication signaling molecules, mediating insect behavioral responses (Howard and Blomquist 2005). Fungal spores can attach to the hydrophobic cuticle using adhesins and/or hydrophobin proteins (Wang and Wang 2017). Degradation of the waxy layer and subsequent cuticular compounds involves the activities of numerous “cuticle-degrading” enzymes, including secreted lipases, proteases, chitinases, and glycosidases (Wang and Wang 2017) (Fig. 2). Degradative products and some components, like hydrocarbons, are metabolized by the fungus via pathways that involve the action of peroxisomes and cytochrome P450 enzymes (Ortiz-Urquiza and Keyhani 2013). Additionally, penetration may involve direct mechanical pressure from growing appressoria (Wang and Leger 2007). Grooming-induced spore removal from the cuticle is likely stimulated by the production of fungal compounds. Extracts of fungal volatiles markedly increase both termite grooming and aggressive behaviors (Yanagawa et al. 2011a). Aggression in some ant and termite species intensifies toward fungus-infected workers, excluding them from other group members (Yanagawa et al. 2011b, Cremer et al. 2018).

Regarding fungal signals potentially recognized and affecting host behaviors, extracellular-associated proteins on the fungus surface induce grooming behavior in *Drosophila* (Shang et al. 2023). Additionally, fungal cell wall components, whether released or secreted, such as β-1,3 glucan, chitin, and secreted proteins, including effectors, hydrophobins, and virulence-related proteases, can potentially activate host insect innate immune pathways before penetrating the host cuticle (Li and Xia 2022). However, whether these compounds affect behaviors such as grooming, burrowing, heat seeking, or molting remains to be determined. The fungal membrane sterol, ergosterol, has emerged as a potentially key fungal molecule recognized by the host, inducing behavioral and immune responses (Rodrigues 2018). *Coptotermes formosanus* termite workers respond to ergosterol levels with increased grooming behavior toward *Metarhizium*-infected nestmates (Chen et al. 2023). In the ant *Linepithema humile, Metarhizium* fungal spores producing lower levels of ergosterol appear to avoid detection by the insect, with the induction of grooming and other sanitary behaviors suppressed (Stock et al. 2023). Altered cuticle components, particularly fatty acids on the host surface used by entomopathogenic fungi during germination, are believed to affect insect behavioral responses (Zhang et al. 2012). It is unclear whether insect compounds, derived from the degradative activities of the infecting fungus and/or released during infection, may stimulate either immunological or behavioral responses.

**Figure 3.**
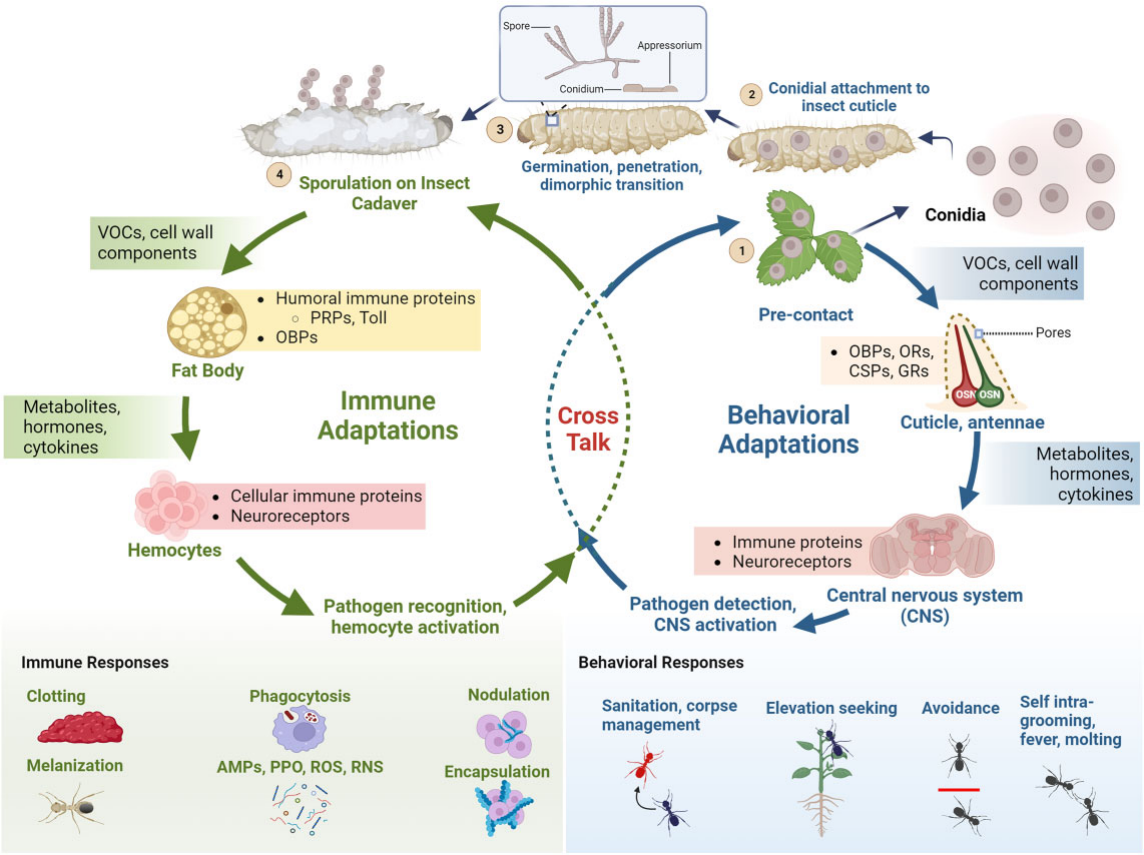
Outline of patterns of cross-talk between behavior and immunity. The behavioral responses of insects induced by fungal infection at different stages of infection can include: avoidance, self/intragrooming, molting, fever, elevation seeking sanitation and hygiene behaviors, and corpse management. Many of these processes are linked to ligand binding/olfactory proteins in the antennae, hemolymph or other tissues, and to neuroreceptors or resident immune proteins in the CNS. Insect immune responses, including melanization, clotting, production of immune effectors (AMPs) hemocyte activation, phagocytosis, nodulation, and encapsulation can be induced by fungal VOCs, cell wall components, and/or metabolites, which act via humoral immune proteins, ligand binding proteins (CSPs and/or OBPs) in the hemolymph and fat body, cellular immune proteins, and receptors in hemocytes and CNS.

After adhesion consolidation, the next fungal infection phase involves germination, appressoria formation, and the initiation of penetration. Detecting and eliminating the pathogen before it reaches the hemocoel, i.e. at the epidermal stage, would represent another line of defense (Pedrini 2018). The epidermis (below the cuticle) and associated cell types, such as oenocytes (also present in the fat body), constitute the outermost aspect of insect tissues (Makki et al. 2014). These cells support various specialized sensory structures extending from the cuticle, such as bristles, hairs, and antennae, and are implicated in immunity (Davis and Engström 2012, Martins and Ramalho-Ortigao 2012). However, little is known about epidermal-level defenses against fungal pathogens aside from their role in supporting cuticle sclerotization and molting, e.g. through regulation and expression of laccases and chitin synthases/chitinases (Merzendorfer and Zimoch 2003, Dittmer and Kanost 2010). Penetrating hyphae from entomopathogenic fungi frequently form close to bristles (Sahayaraj et al. 2014). This may be because the cuticle’s thinner components make these areas easier to penetrate. However, *Drosophila* mechanosensory bristles exhibit nonselective detection of dust, including fungal spores, landing on the fly, which can then trigger grooming/cleaning behaviors (Zhang et al. 2020a). In the early stages of fungal infection, specific olfactory-related proteins in the insect host can show significant differential expression (Levy et al. 2004, Zheng et al. 2021, Zhang et al. 2021a). A mutant of the *Drosophila* odorant receptor coreceptor, ORCO, results in the inability of the fly to recognize chemicals associated with eliciting grooming behavior (Yanagawa et al. 2018). Additionally, the *Drosophila* chemosensory protein CheA75a has been shown to recognize the *Metarhizium robertsii* spore surface, specifically the fungal extracellular membrane protein Mcdc9, inducing self-hygienic behavior that involves the removal of fungal spores from the insect body (Shang et al. 2023). In tsetse flies, two odorant-binding proteins, OBP6 and OBP28a, are upregulated during symbiont (*Wigglesworthia*) colonization, influencing melanization and cellular immunity through various insect transcription factors, including *lozenge* (Benoit et al. 2017). Apart from olfactory proteins, locusts, which can transition between solitary and gregarious social lifestyles, activate the upstream immune modulator Gram-negative binding protein 3 in the gregarious lifestyle, likely establishing prophylactic immunity and reducing fungal spread, suggesting a role for pattern receptor proteins in both behavior and immunity (Wang et al. 2013). Similarly, two compounds secreted by termite salivary glands, termicin and Gram-negative binding protein 2, exhibit antifungal activity (Hamilton and Bulmer 2012). These data support the idea that insects can detect and respond to fungal components such as VOCs via specific chemoperception (olfaction) or immunity-recognizing proteins to affect behavioral and immune communication during fungal contact with the host (Fig. 3).

## Cross-talk: during the internal growth stage of entomopathogenic fungi in insects

After penetrating the integument and entering the hemocoel, in most cases examined thus far, the infecting fungus undergoes a dimorphic transition, from hyphal growth to the production of free-floating single-celled hyphal bodies. These cells travel within the insect (open) circulatory system, using hemolymph nutrients and accessing/infecting internal tissues that can include the tracheae, muscles, digestive tract, and reproductive organs (Toledo et al. 2010). During this *in vivo* growth stage, various innate immune responses in the insect are activated. The fat body and hemocytes mediate humoral and cellular processes that are part of insect innate immunity. These processes have well-described responses to a variety of microbes, including fungi (Lemaitre and Hoffmann 2007). Humoral immune-related pathway components, such as those of the pattern recognition receptors, Toll, IMD, and JAK/STAT pathways, and immune-related factors, including AMPs, lysozymes, apolipophorin III, hemocyanin, and transferrin, are produced in insects in response to fungal infection (Eleftherianos et al. 2021). Host cellular immunity includes coagulation, hemocyte activation and phagocytosis, encapsulation, melanization, prophenoloxidase cascade, and oxidative burst production, which have been well-characterized as antimicrobial responses against fungi at this stage of infection processes (Li and Xia 2022). However, entomopathogenic fungal hyphal bodies have evolved various strategies to evade the insect innate immune system, including escaping encapsulation and phagocytosis, masking the fungal cell surface to avoid detection, and producing toxins and other compounds that suppress the host immune reaction (Huang et al. 2023). Indeed, *Metarhizium* and *Beauveria* can grow within insect cells as well as inside soil predatory amoeba, suggesting links between insect pathology and survival in soils (Kurtti and Keyhani 2008, Bidochka et al. 2010). As fungal cells proliferate within the hemocoel, the host immune system is overpowered by the activity of the fungus and fungal proliferation. In addition to innate immunity, behavioral alterations are typically observed during this infection stage, thereby affecting feeding, aggression, response to semiochemicals, and reproductive behavior. Notably, certain fungal entomopathogens, particularly those with narrow host ranges, induce characteristic behavioral alterations aimed at increasing transmission. Host “zombification” can lead to diverse behaviors, from height seeking followed by mandibular fixation (as observed in ants infected by *Ophiocordyceps* and flies infected by *Entomophthora*) to increased or frenzied mating (shown in cicadas infected by *Massospora*) (Macias et al. 2020, de Bekker et al. 2021, Elya and De Fine Licht 2021).

Fungal PAMPs, cell wall components, proteins, effectors, metabolites, and even miRNAs have been shown to be secreted by fungal pathogens, along with other uncharacterized factors that can either activate and/or facilitate evasion of host chemoperception, innate immunity, and antifungal behaviors (Qu and Wang 2018) (Fig. 2). Strategies employed by the fungus to shield itself from host defenses include the production of a collagenous coat within the hemocoel (Wang and Leger 2006), evasion of host innate immunity via antioxidant enzyme production, and cell wall protein and glucan rearrangements (Lu and Leger 2016). A fungal laccase secreted during this stage also functions in innate immunity (Lu et al. 2021). Moreover, various entomopathogenic fungi produce an array of secondary metabolites during growth in the insect hemocoel. These include products of nonribosomal polyketide synthetases and secondary metabolite cluster pathways (Gibson et al. 2014). In *Metarhizium*, destruxins, a group of cyclohexadepsipeptides, exhibit diverse insect toxic effects, including the inhibition of humoral immune pathways (such as Toll/Imd), reduction of AMP production, inhibition of host prophenoloxidase and oxidative burst responses, and cytotoxicity to hemocytes and other cells (Hu et al. 2016). Destruxins have immunosuppressive effects, including cellular immune system dysregulation in insects, preventing nodule formation, and disrupting phagocytosis (Vey et al. 2002). They also affect Ca^2+^ influx, decrease intracellular free H^+^ levels, affect muscle function, and induce paralysis and sluggishness (Ruiz-Sanchez et al. 2010). Furthermore, these toxins can induce cell apoptosis through increased oxidative stress and interfere with mitochondrial and hormonal signaling pathways, as well as acetylcholine receptors (Butt et al. 2016). Certain destruxins serve to inhibit behavioral fever (Hunt and Charnley 2011), and mutants that produce enhanced levels of destruxins have been shown to be more virulent (Huang et al. 2022, Li et al. 2022). Additionally, other toxic metabolites isolated from various insect pathogenic *Beauveria, Metarhizium*, and *Isaria* species, such as beauverolides, beauvericin, tennelin, isarolides, brassinolide, cytochalasins, and oosporein, exhibit diverse behavioral and immune effects (Sinha et al. 2016). These findings highlight the important role of secondary metabolite toxins in potentially mediating cross-talk between the antifungal behaviors and the antifungal immunity.

As a consequence of infection, host-derived metabolites also undergo marked changes in content and concentration as the fungus grows inside the host. These changes are driven in part by fungal degradation and consumption of host tissues, including alterations in carbohydrate, protein/peptide/amino acids, fatty acid, lipid, arachidonic acid, and amine profiles, as well as by host responses to the invading fungus (Xu et al. 2015, Zhang et al. 2023b) (Fig. 3). In certain instances, different metabolites and degradative products can feed into the regulatory pathways that control innate immune responses. For example, entomopathogenic fungi can suppress host innate immunity by inhibiting the production of host arachidonic acid during infection (Dean et al. 2002). Interestingly, immune activation components, such as the microbial cell wall components laminarin and lipopolysaccharide, can induce behavioral fever in locusts (Bundey et al. 2003). Eicosanoid biosynthesis is implicated in this response, likely regulating the transformation of arachidonic acid to prostaglandins, with the latter controlling body temperature in both mammals and arthropods (Bundey et al. 2003). These data establish aspects of the biochemical link between behavioral and immunological responses, i.e. mediated by either the presence or detection of microbial components and/or circulating insect and fungal metabolites (e.g. eicosanoids). Tryptamine production by *M. robertsii* has been shown to induce the expression of immune signaling-related genes and reactive oxygen species production in grasshoppers via the IMD and Toll pathways by activating the host aromatic hydrocarbon receptor LmAhR (Tong et al. 2020); however, this inhibits the expression of the AMP defensin, thus tryptamine production by the fungus acts to suppress host antifungal responses. In plants, tryptamine functions in repressing herbivore feeding and reproduction (Gill et al. 2003). Additional fungal components, including effector proteins that are secreted and target specific host proteins (e.g. signal pathway-interfering toxins, serine proteases, eckinase, and tyrosine phosphatase) and secondary metabolites (e.g. nonribosomal peptide and polyketide mycotoxins, destruxins, and alkaloids), have been shown to affect insect behavior, with such effects primarily explored in terms of antifeedant and lethargic activities (Gibson et al. 2014, Will et al. 2020, Toopaang et al. 2023).

In addition, during the internal growth phase of the fungal pathogen in host insects, the expression of various neuronal-related genes in the CNS, particularly those associated with morphogenesis, development, behavior, cognition, learning, and memory, is significantly altered (Zhang et al. 2017) (Fig. 3). The consequence of these changes remains unknown but may either involve (i) behavioral attempts to hinder infection (such as heat-seeking or grooming), (ii) neurological impairment and/or degradation due to extensive fungal growth, and/or (iii) pathogen efforts to manipulate host behavior in order to benefit sporulation and/or dissemination of the pathogen upon host death. Some of the differentially expressed genes may be involved in both behavioral and immune functioning, with modulation of neuronal signaling occurring during infection. For example, the octopamine and dopamine receptors (*Dop1R1*) in *Drosophila*, known to function in the formation of appetitive memories for sugar and other foods, play a role in learning (Burke et al. 2012). Octopamine, with contrasting immunoenhancing and immunosuppressive effects, is a key hormone in acute stress responses related to flight or fight behavior, and injecting locusts with octopamine has been shown to increase their susceptibility to *M. anisopliae* (Goldsworthy et al. 2005). Given that both neurons and immune cells have octopamine receptors, octopamine and dopamine ligand/receptors may contribute to cross-talk between behavioral and immune responses. Another example is the upregulation of the activated metabotropic glutamate receptor 2/3 in *M. acridum*-infected locusts at different fungal infection time points, suggesting the existence of a mechanism for CNS sensing of infection and induction of neuroprotective pathways (Zhang et al. 2017). Additional affected neurological-related processes include the expression of glutaminase, implicated in immunological challenge protection, and calmodulin, involved in inflammation, promoting immune signaling by controlling nitric oxide production (Zhang et al. 2017).

The CNS itself includes resident immune surveillance mechanisms, such as microglia and Toll-like receptors (Fig. 3). However, the roles and responses of these mechanisms in fungal-mediated infection remain to be explored. γ-Aminobutyric acid type A receptors expressed on T cells inhibit the cell responses to antigens (Tian et al. 1999). The reported decrease in γ-aminobutyric acid type A expression during infection in locusts may inhibit such immune responses, indicating a potential link to immunity in both the CNS and immune tissues. Regarding links to chemoreception, little evidence exists; however, environmental odors can influence immune components, e.g. during hematopoiesis. Sensing food odors via the Or42a odorant receptor in *Drosophila* stimulates projection neurons (Asahina et al. 2009), leading to downstream activation of neurosecretory cells, which in turn mediates the release of GABA into the hemolymph, potentially functioning in immunity.

## Cross-talk: pre- and postmortem

In several systems, such as “zombie” ants, infected insects exhibit elevation-seeking behaviors considered to aid in the dispersal of emerging fungal spores (de Bekker et al. 2021). Social insects, notably ants, termites, and honeybees, display various forms of sanitation behavior toward sick and deceased nest mates, involving self-removal, conspecific removal, burial, or dismemberment of dead or dying nestmates (Sun and Zhou 2013). Additionally, fungal antagonistic microbes that produce antifungal compounds may be recruited, constituting a form of “biological warfare” (Mattoso et al. 2012, Hong et al. 2023b). Dying bees, for instance, actively self-exclude from the hive, presumably to reduce the risk of infecting nestmates (Rueppell et al. 2010). In the garden ant, *Lasius neglectus*, workers enhance brood care and hygienic behaviors in the presence of *M. anisopliae*-contaminated workers (Ugelvig and Cremer 2007). These behaviors seek to limit fungal growth in cadavers and reduce the likelihood of infection of healthy nestmates. This can be particularly important as the fungal spores produced on cadavers show greater virulence compared with those isolated from artificial media, suggesting optimization of virulence-related processes in the presence of specific hosts (Hussain et al. 2010). Additionally, the fungus produces specific compounds, such as oosporein by *B. bassiana*, that help maximize cadaver utilization by suppressing competing microbes (Fan et al. 2017).

Various compounds induce cleaning and hygienic behaviors in insects. For example, phenethyl acetate, 2-phenylethanol, and benzyl alcohol released by fungal-infected bees induce nest cleaning behavior (Swanson et al. 2009). Fungal infection of ants, including pupae, leads to alterations in surface fatty acids, which then act as chemical signals to induce cadaver removal behaviors (Qiu et al. 2015). Similarly, octanol, octanone, and other compounds released from dead termite cadavers have been shown to induce corpse removal (Sun et al. 2017). Some chemicals not only modulate behavior but also function in insect innate immunity, e.g. 2-phenylethanol production by *Metarhizium* inhibits AMP production in locusts (Zhang et al. 2023a). The VOC profiles of entomopathogenic fungi cultivated in artificial culture and on insect cadavers differ (Hussain et al. 2010), indicating potential variations in effects on behavioral and immune responses based on growth substrates. Further exploration is needed to understand these sanitation and hygienic behaviors, including identifying critical chemicals and genetic pathways that modulate these behaviors and determining their relationship with innate immunity.

## Future challenges

The interaction between entomopathogenic fungi and insects occurs on spatial and temporal scales, involving strategic maneuvers from both participants. Spanning initial contact, germination, penetration, dimorphic transition, internal proliferation, extension outward, and sporulation on the cadaver, this dynamic interaction occurs over hours, days, or longer. This permits substantial levels of selection and coevolutionary relationships to develop, intertwining insect behavior and innate immune responses. These are likely dual strategies that determine whether hosts succumb to pathogen infection or overcome such infections (Fig. 3). During the early phases of the interaction, before the hemocoel produces hyphal bodies, insects primarily use behavioral defenses, like avoidance or grooming, to stop the infection from spreading. These early-stage behavioral defenses are vital for social insects in minimizing exposure, potentially resulting from the success of their social adaptations in nature. Evidence suggests the initiation of innate immunity activation as early as possible, i.e. prior to cuticle penetration, can function to “prime” the organism for combating infection (Zhang et al. 2020b). Detection and response to fungal compounds (e.g. cell wall components, secreted products, and VOCs) likely undergo strong selection. Indeed, investing in behavioral defenses becomes a strategic approach to preventing infection and reducing reliance on innate immunity. The insect lifestyle markedly influences the balance between behavioral and innate immunity mechanisms, with soil-dwelling and social insects favoring behavioral adaptations (such as sanitation), whereas solitary or more mobile insects may opt for avoidance strategies (Meunier 2015). Further examination of these issues is likely to yield novel information on the evolutionary strategies that have emerged in response to fungal infection. As fungal penetration occurs, the options available to the insect become constrained. Some insects may attempt additional behavioral responses, such as grooming or heat seeking, whereas others invest in resistant physical barriers (such as the epicuticle) to limit water and nutrient availability and potentially as a reservoir for antiseptic compounds (Ortiz-Urquiza and Keyhani 2013). Further research is needed to determine whether developmental responses such as shedding of the cuticle or early pupation are positive or passive strategies that contribute to reducing pathogen infection and/or increasing survival.

Additional avenues of research include addressing the ecological roles and evolutionary mechanisms of cross-talk between immunity and behavior, particularly before physical contact and during initial spore attachment to the cuticle. Unresolved questions include why the fungus would produce VOCs that lead to insect avoidance, potentially reducing their chances of finding a host, i.e. what role do these VOCs play naturally, and/or whether some entomopathogenic fungi have evolved mechanisms to mask VOCs that repel hosts. One aspect that remains poorly examined is that this phenomenon is likely impacted within the context of plant associations. In this case, plants may seek to emit VOCs to repel them, but the consequence of the fungal–plant association on such activities remains unknown (Branine et al. 2019, Thompson 2022). Entomopathogenic fungi are proposed to have evolved from grass endophytes ~100 million years ago, and the differentiation or coselection from endophytic colonization is suggested to have helped promote the acquisition of insect pathogenic genes (Quesada Moraga 2020). One hypothesis is that entomopathogenic fungi with improved pathogenicity result in plant protection from herbivorous insects and access to previously nonutilizable nutrient resources, and in exchange, the fungus obtains carbon within a semiprotected ecological niche (i.e. rhizosphere, epiphyte, and/or endophyte) (Iwanicki et al. 2022). Thus, the selection for maintaining and/or enhancing the ancestral fungal plant remains strong. Genetic mechanisms, including horizontal gene transfer (from plant to fungus and/or from insect to fungus), may facilitate the emergence of pathogens with broad host ranges from ancestors with narrow and restricted host ranges (Zhang et al. 2019). Given the large diversity and distribution of fungal entomopathogens, it is important to consider both convergent and divergent evolutionary mechanisms (Arnesen et al. 2018).

Once the fungus has reached the hemocoel, morbidity becomes almost inevitable. Behavioral responses, such as ingesting antimicrobial components or dietary modification, can support immune defenses (Leal et al. 2022), but at this advanced stage, the infection process likely affects insect behavior, with innate immune defenses becoming less effective. Chitin (including variously deacetylated forms and, to a lesser extent, chitosan) acts as an important component due to its central role in the structure of insect cuticles and presence in the fungal cell walls, with chitin oligosaccharides also found in plants (Sánchez-Vallet et al. 2015, Pusztahelyi 2018, Moussian 2019). Chitin functions as a microbe-associated molecular pattern in plant pathogenic fungi and is recognized as a nonself-component by plant lysin motif (LysM)-containing receptor kinases. This recognition triggers host immune responses during microbe–plant interactions (Hu et al. 2021). LysM proteins are also found throughout the fungal kingdom, including plant and insect pathogens, and are thought to be produced by pathogenic fungi to mask their cell wall chitin from detection by host (whether plant or insect) defense systems (Akcapinar et al. 2015). Functional aspects of LysM effectors in entomopathogenic fungi have been characterized; e.g. a family of 12 LysM proteins is found in *B. bassiana*. These proteins have been shown to suppress chitin-induced insect immunity, help protect the fungal cells from phagocytosis and nodulation, and hence act to promote fungal virulence. Another strategy involves deacetylation of chitin oligomers, as seen in a number of fungal plant pathogens, which helps the fungus evade recognition by host chitin receptors, allowing for survival in host plants (Sánchez-Vallet et al. 2015). Similarly, the deacetylation of chitin in the entomopathogenic fungus *B. bassiana* has been recently reported to contribute to fungal virulence (Liu et al. 2023b). However, the role of chitin in mediating the cross-talk between behavioral and immune responses in fungal–insect interactions remains unknown.

Following the death of a host, healthy insects either avoid or remove the corpses, especially in the case of social insects. This corpse management and the various strategies employed can significantly impact pathogen dispersal and insect prophylaxis. In response, certain entomopathogenic fungi are known to hijack host behavior to increase transmission (de Bekker et al. 2021). A prime example of this is the host-specific parasitic “zombie”-ant fungus, *Ophiocordyceps unilateralis* (*sensu lato*), which manipulates ant behavior to facilitate fungal development and spore dispersal, a phenomenon known as the fungal extended phenotype (Andersen et al. 2009, Hughes et al. 2011a). Zombie ant fungi may target host phototaxis in a circadian manner to increase locomotor activity (Andriolli et al. 2019), causing the ant to deviate from foraging trails and exhibit convulsions and twitches as it attempts to climb foliage (elevation seeking) (Pontoppidan et al. 2009, Hughes et al. 2011a, de Bekker et al. 2015). The invasion of mandibular muscle tissues causes the ant to cling to the vegetation (Pontoppidan et al. 2009, Hughes et al. 2011a), resulting in a final “death grip” or manipulated bite that causes mandibular muscle atrophy, locking the jaw and preventing the cadaver from falling (Hughes et al. 2011a). Fungal growth during the ant’s death forms a sexual structure from which fresh spores are released, initiating a new infection cycle (de Bekker et al. 2015). Fossil evidence dating back to 48 million years suggests that this manipulated behavior of ants can be traced back to the Eocene (Hughes et al 2011b). Certain aspects of the *Ophiocordyceps*-ant infection cycle remain unclear due to the uniqueness and complexity of this model system (de Bekker 2019). Nevertheless, it has been suggested that enterotoxins, aflatrem, and other potential fungal effectors that disrupt ant feeding behaviors could be linked to differentially expressed genes associated with circadian rhythms, clock-controlled genes, odor detection pathways (odorant receptors, OBPs), and neurotransmitter signaling (kynurenic acid, biogenic monoamines, and dopamine) (Will et al. 2020, de Bekker et al. 2021), and many of these genes may have dual functions in both behavior and immunity.

## Concluding remarks

Current and future directions linking fungal infection stages with specific olfactory or immune components that may then function/elicit both behavioral and immunological responses are likely to yield important insights into largely unexplored aspects of the infection process. Although each stage appears to have distinct behavioral and immune-related responses, the links between these have only begun to be elaborated. In the longer term, these studies are likely to shed important fundamental insights into the biology of the interaction between these organisms and can help improve strategies for exploiting entomopathogenic fungi in pest control. Such applications, either as an alternative to chemical pesticides and/or as part of integrated pest management practices, can be useful by targeting specific innate or behavioral immune pathways in host-specific ways that would help minimize nontarget effects, resulting in more environmentally friendly and effective means of eliminating noxious insect pests.
